# *HES5* silencing is an early and recurrent change in prostate tumourigenesis

**DOI:** 10.1530/ERC-14-0454

**Published:** 2015-04

**Authors:** Charles E Massie, Inmaculada Spiteri, Helen Ross-Adams, Hayley Luxton, Jonathan Kay, Hayley C Whitaker, Mark J Dunning, Alastair D Lamb, Antonio Ramos-Montoya, Daniel S Brewer, Colin S Cooper, Rosalind Eeles, UK Prostate ICGC Group, Anne Y Warren, Simon Tavaré, David E Neal, Andy G Lynch

**Affiliations:** 1 Cancer Research UK Cambridge Institute, University of Cambridge, Cambridge, CB2 0RE, UK; 2 Division of Genetics and Epidemiology, The Institute of Cancer Research, Sutton, UK; 3 Department of Biological Sciences and School of Medicine, University of East Anglia, Norwich, UK; 4 Royal Marsden NHS Foundation Trust, London and Sutton, UK; 5 Department of Pathology, Addenbrooke's Hospital, Hills Road, Cambridge, CB2 2QQ, UK; 6 Department of Urology, Addenbrooke's Hospital, Hills Road, Cambridge, UK; 7 Department of Surgical Oncology, Addenbrooke's Hospital, University of Cambridge, Hills Road, Cambridge, CB2 2QQ, UK

**Keywords:** prostate cancer, epigenetics, methylation, HES5, HES6, AR, ERG, NOTCH

## Abstract

Prostate cancer is the most common cancer in men, resulting in over 10 000 deaths/year in the UK. Sequencing and copy number analysis of primary tumours has revealed heterogeneity within tumours and an absence of recurrent founder mutations, consistent with non-genetic disease initiating events. Using methylation profiling in a series of multi-focal prostate tumours, we identify promoter methylation of the transcription factor *HES5* as an early event in prostate tumourigenesis. We confirm that this epigenetic alteration occurs in 86–97% of cases in two independent prostate cancer cohorts (*n*=49 and *n*=39 tumour–normal pairs). Treatment of prostate cancer cells with the demethylating agent 5-aza-2′-deoxycytidine increased *HES5* expression and downregulated its transcriptional target *HES6*, consistent with functional silencing of the *HES5* gene in prostate cancer. Finally, we identify and test a transcriptional module involving the AR, ERG, HES1 and HES6 and propose a model for the impact of *HES5* silencing on tumourigenesis as a starting point for future functional studies.

## Introduction

Current analysis of cancer genome sequencing has revealed disease processes and genomic alterations that may underlie disease initiation or evolution (Nik-Zainal *et al*. 2012, Baca *et al*. 2013, Tarpey *et al*. 2013). These approaches have identified and enumerated recurrently mutated driver genes in several cancer types, such as *KRAS* which is mutated in 93% of pancreatic cancers (Biankin *et al*. 2012) and *TP53* which is mutated in 96% of high-grade serous ovarian cancers (Cancer Genome Atlas Research Network 2011), 69% of oesophageal cancer (Weaver *et al*. 2014) and over 50% of colorectal cancers (Cancer Genome Atlas Network 2012). In contrast with these highly recurrent mutations, a recent study of 112 aggressive primary prostate cancers has reported that the most significantly mutated gene (*SPOP*) was altered in only 13% of cases, with the next most commonly affected gene *TP53* affected in only 6% of prostate tumours (Barbieri *et al*. 2012).

Therefore, while genome sequencing approaches have provided important insights into the biology of prostate cancer (Berger *et al*. 2011, Baca *et al*. 2013, Lindberg *et al*. 2013, Weischenfeldt *et al*. 2013) the high intra- and inter-tumour heterogeneity coupled with the small samples sizes may have limited the identification of genetic driver events in primary tumours. Indeed, previous genome sequencing studies have reported few common mutations between different tumour foci within the same prostate (Lindberg *et al*. 2013), highlighting marked intra-tumour heterogeneity and the absence of a genetic founder mutation. This complexity has led many groups to focus on late-stage, aggressive disease with the aim of identifying genomic events associated with disease progression (Barbieri *et al*. 2012, Grasso *et al*. 2012). However, their remain important unanswered questions over the early stages of prostate tumour evolution where genetic events appear to be for the most part heterogeneous. One notable exception to this is gene fusions involving ETS (E26 transformation-specific) transcription factors that have been found to occur in approximately half of all prostate cancers (Tomlins *et al*. 2005, Perner *et al*. 2006). However, these androgen receptor (*AR*)-driven gene fusions alone are insufficient to initiate prostate tumours in disease models (Carver *et al*. 2009, Chen *et al*. 2013) and may not be early ‘founder’ events in disease evolution (Barry *et al*. 2007, Mertz *et al*. 2013, Minner *et al*. 2013).

Therefore current evidence would seem to suggest that if a common initiating driver event exists it is not genetic, implicating other mechanisms in disease aetiology. In addition to somatic mutation several other disease-initiating pathways have been proposed in prostate cancer including germline predisposition (Kote-Jarai *et al*. 2011, Eeles *et al*. 2013), telomere shortening (Sommerfeld *et al*. 1996, Heaphy *et al*. 2013), chronic inflammation (Elkahwaji *et al*. 2009, Caini *et al*. 2014), metabolic stress (Freedland 2005, Kalaany & Sabatini 2009) and epigenetic alterations (Lee *et al*. 1994, Kanwal *et al*. 2014). It is likely that non-genetic and genetic alterations interact during tumourigenesis and several studies have identified interactions between somatic mutations and micro-environmental changes (Garcia *et al*. 2014), inflammation (Kwon *et al*. 2014) and metabolism (Kalaany & Sabatini 2009). Current technologies allow accurate identification and quantification of epigenetic alterations and are therefore a tractable second line of enquiry to identify driver events in prostate tumourigenesis.

We have recently identified a role for the enhancer of split transcription factor *HES6* in prostate cancer and AR signalling (Ramos-Montoya *et al*. 2014). Herein, we characterise an epigenetic alteration at the promoter of the related *HES5* gene, which has been recently reported in a panel of genes that showed promise as a prostate cancer marker in biopsy samples (Paziewska *et al*. 2014). We profile this change in detail and show it to be an early event in prostate cancer development and highly recurrent across three unrelated prostate tumour cohorts. We then characterise an interaction between the epigenetic silencing of *HES5* and the expression of *HES6* and provide evidence for interactions with known oncogenic pathways in prostate cancer (namely AR signalling and *ERG* gene fusions), highlighting a transcriptional network that is altered in prostate cancer development first by an epigenetic change and then by a genomic rearrangement.

## Materials and methods

### Sample cohorts

**Figure 1:**
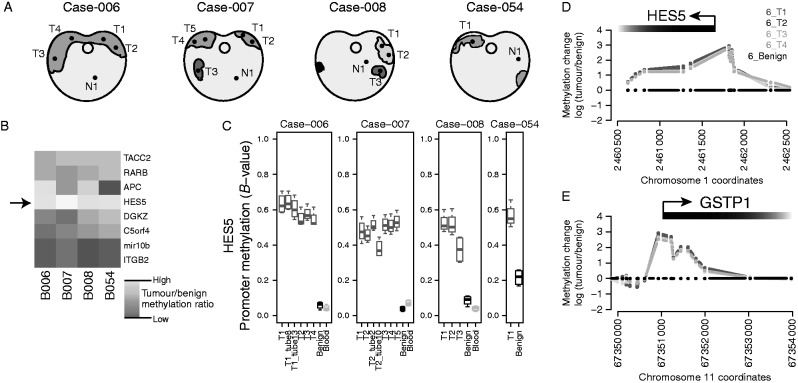
*HES5* promoter methylation is an early event in prostate tumourigenesis. (A) Representation of sections through four cancerous prostates from which multiple tumour cores (T1–T5) and adjacent benign cores (N1) were taken for methylation analysis. Regions in purple indicate histologically malignant foci and different shades of purple indicate tumour foci that appeared unconnected in 3D-sectioning. Sample keys provided are ICGC Prostate UK IDs. (B) Heatmap showing the median tumour over benign methylation changes at regions in the promoter regions of eight candidate genes. (C) Boxplots showing the methylation status at the promoter region of *HES5* in the cohort of prostate tumours with multiple tissue cores, adjacent benign and blood DNA samples. Boxplots depict quartiles for probes within promoter region genomic windows, error bars denote 95% CI and data points are shown for values outside 95% CIs. (D and E) Genomic views of DNA methylation in tumour cores compared with adjacent benign tissue for (D) the *HES5* gene promoter region and (E) the methylation-positive control *GSTP1* gene promoter. Plots show the methylation profiles from multiple tumour foci for Case-006, data are presented as log_2_ ratio of tumour over benign. Gene promoters and orientation are annotated at the top of each plot.

In a series of four radical prostatectomy specimens, we systematically dissected the whole prostates, identified regions containing tumour and harvested 17 tumour-rich samples from 13 spatially separated tumour cores (median 46% tumour, interquartile range (IQR) 36–62%), four adjacent benign samples and three whole-blood samples (Fig. 1a and Supplementary Figure 1a, see section on supplementary data given at the end of this article). Each tumour core was taken from a 5 mm tissue slice and the tumour content of samples used for DNA extraction was assessed by a pathologist using H&E staining of immediately adjacent sections (Warren *et al*. 2013). From two such cores, we also took three sets of sections for DNA extraction to allow assessment of heterogeneity within cores in addition to the spatial heterogeneity within and between cancerous prostates (Supplementary Figure 1a). These samples were used for global methylation profiling using Infinium HumanMethylation450 arrays (see below for details).

In a separate cohort of 39 matched prostate tumour and adjacent benign samples, we performed targeted bisulphite sequencing of the *HES5* promoter, to assess the frequency of *HES5* hypermethylation in prostate cancer. This analysis provides a promoter-wide view of DNA methylation changes at the *HES5* promoter (in contrast to the limited number of CpGs assessed using methylation profiling arrays).

In an unrelated, larger cohort of prostate cancers with publicly available methylation array data (*n*=304 tumours, *n*=49 matched normal samples) (Weinstein *et al*. 2013), we assessed the recurrence of *HES5* promoter methylation.

### DNA methylation profiling in blood, benign prostate and multiple spatially separate tumour foci

Clinical samples for analysis were collected from prostatectomy patients with full research consent at the Addenbrooke's Hospital, Cambridge, UK. The prostates were sliced and processed as described previously (Warren *et al*. 2013). A single 5 mm slice of the prostate was selected for research purposes. Tissue cores of 4 mm or 6 mm were taken from the slice and frozen. The frozen cores were mounted vertically and sectioned transversely giving a single 5 μm frozen section for H&E staining followed by 6×50 μm sections for DNA preparation using the Qiagen Allprep kit. Using the Infinium HumanMethylation450 BeadChip kit, DNA was subjected to bisulphite conversion, amplification, fragmentation, hybridisation, extension and labelling, according to the manufacturer's instructions (Illumina, Little Chesterford, Essex, UK). Bead summary data from Infinium HumanMethylation450 arrays were processed using the Minfi package in the R statistical software (Aryee *et al*. 2014, R-Core-Team 2014). As previously described, probe types were normalised separately (Marabita *et al*. 2013) before generating *M*- and *B*-values for exploratory analysis. Summary plots were generated in the R statistical software (R-Core-Team 2014). Raw and processed data have been uploaded to the ArrayExpress portal under accession E-MTAB-2964, in addition all code used to generate figures in the paper are included as part of the R-markdown HTML document available on our group webpage.

### Targeted bisulphite sequencing

PCR primers were designed to amplify a 441 bp fragment from the *HES5* promoter containing 60 CpGs (*HES5*-BSx-F: 5′-GAGGGGGTGTTAGGTTGGTT-3′; *HES5*-BSx-R: 5′-ACCCACCTACTCCTTAAAAAAC-3′). The amplicons were generated separately for 39 matched tumour normal sample pairs and assessed before preparing barcoded sequencing libraries using a Nextera XT kit (Illumina). Barcoded DNAs were quantified and equal amounts of each indexed library were then pooled and sequenced on an Illumina MiSeq (PE300). Fastq data files were split using index sequences and downstream methylation analysis was performed using Bismark (Krueger & Andrews 2011) and summary plots and test statistics were generated using the R statistical software (R-Core-Team 2014). This analysis gave a median sequencing coverage of 786× (Supplementary Figure 3, see section on supplementary data given at the end of this article). All code used to generate figures in the paper are included as part of the R-markdown HTML document available on our group webpage.

### Data mining

An R markdown document containing all code required to reproduce our analysis and all figures has been included as a supplementary HTML document (available on our group webpage). Briefly, DNA methylation 450k array data for LNCaP prostate cancer cells and PrEC benign prostate epithelial cells (CC-2555, Lonza, Basel, Switzerland) were obtained from GEO (triplicate data for each cell line from GSE34340 and singleton data for each cell line from GSE40699) (Statham *et al*. 2012, Varley *et al*. 2013) and summary plots were generated using the R statistical software (R-Core-Team 2014). Gene expression data from LNCaP cells treated with the demethylating agent 5-aza-2′-deoxycytidine were retrieved from GEO (GSE25346). Gene expression data from human prostate benign and tumour tissues were obtained from GEO (GSE3325). Gene expression data from control and ERG-knockdown VCaP cells was retrieved from GEO (GSE60771). All GEO data were retrieved using the GEOquery package in the R statistical software and summary plots were generated using the same software (Davis & Meltzer 2007, R-Core-Team 2014). Transcriptional networks were drawn using the BioTapestry application (Longabaugh 2012) constructing models using ChIP-seq binding profiles, expression correlations and published transcriptional links.

### HES5 motif enrichment analysis

The position weight matrix for HES5 was obtained from Yan *et al*. (2013) and used to search the genomic sequence of the *HES6* gene locus (including 1 kb upstream and 1 kb downstream sequence). Motif searches were carried out using the RSAT matrix-scan (with human ‘upstream-noorf’ background control) (Turatsinze *et al*. 2008), and motif scores were visualised using BioSAVE (Pollock & Adryan 2008).

### Androgen time-course gene expression profiling in LNCaP and VCaP cells

Following 72-h steroid depletion in the media containing 10% charcoal-stripped FBS, LNCaP and VCaP cells were subjected to androgen stimulation (1 nM R1881) or vehicle control treatment (0.01% ethanol). The cells were harvested at the indicated timepoints over a 24 h period following treatment and RNA extracted using Trizol (Life Technologies). For the LNCaP treatment time-course, a full analysis has been published (Massie *et al*. 2011) and raw and normalised data have been deposited at GEO (GSE18684). Data for the VCaP androgen treatment time-course have also been deposited at ArrayExpress (E-MTAB-2968). Expression data were analysed using the beadarray software, with spatial artefacts identified and removed automatically (BASH) and curated manually (Dunning *et al*. 2007, Cairns *et al*. 2008). The resulting data set was summarised with outliers removed to obtain mean log-intensity and standard error for each probe/array combination.

## Results

### *HES5* promoter methylation is an early event in prostate tumourigenesis

In order to investigate the epigenetic landscape within and between prostate tumours, we systematically dissected four radical prostatectomy specimens, harvesting 17 tumour-rich samples from 13 spatially separated tumour cores (median 46% tumour, IQR 36–62%), four adjacent benign samples and three whole-blood samples (Fig. 1a and Supplementary Figure 1a). Consistent with previous reports (Lindberg *et al*. 2013), these spatially separated tumour cores appeared to be only distantly related by somatic mutations and therefore our aim was to identify early (common ‘trunk’) epigenetic events. Analysis of the methylation distributions for all assayed CpGs revealed that global methylation profiles were similar between tumour and benign prostate samples (Spearman's rank correlation of tumour vs benign methylation profiles 0.94–1.00; Supplementary Figure 1b,c, d and e). A recent study has highlighted eight genomic loci that showed differential methylation in a series of unmatched tumour and benign prostate samples (i.e. from different individuals), a subset of which were proposed as molecular markers to support pathological diagnosis of biopsies (Paziewska *et al*. 2014). We assessed the reproducibility and clonality of these eight differentially methylated regions in our cohort of cases with multiple spatially separate tumour samples, matched benign tissue and blood DNA samples (Fig. 1b and Supplementary Figure 1f, g, h, i, j, k, l, m).

In our cohort, the promoter region of the *HES5* gene showed the largest and most consistent increase in methylation in tumour samples compared with matched normal tissue (median 7.6-fold increase, median variance=0.003), together with consistently low methylation in adjacent normal tissue (median normal methylation=0.08, median variance=0.0006; Fig. 1b, c, d and Supplementary Figure 1f, g, h, i, j, k, l, m). The study by Paziewska *et al*. (2014) showed low *HES5* promoter methylation in benign prostatic hyperplasia and hypermethylation in prostate tumour biopsies. Among the other regions examined, we found that tumour methylation at the ITGB2 and mir10B loci showed no difference with matched benign tissue, the APC locus showed variable differences between tumour and matched benign and the remaining four loci (*RARB*, *C5orf4* (*FAXDC2*), *TACC2* and *DGKZ*) showed increased methylation in tumour vs matched benign samples, although to a lesser extent than the *HES5* locus (Fig. 1b, c, d and Supplementary Figure 1f, g, h, i, j, k, l, m). The tumour-specific methylation changes at the *HES5* promoter were consistent within and between cases and comparable with the hypermethylation observed at the *GSTP1* gene (Fig. 1d and e), which is invariably silenced in prostate cancer and has been extensively studied (Lee *et al*. 1994). These consistent methylation changes at the *HES5* promoter appear to be locus specific, as highlighted by the similarity of global methylation profiles (Supplementary Figure 1b, c, d and e) and the absence of consistent changes in DNA methylation at other genomic loci across spatially separated tumour samples from the same patient (Supplementary Figure 2, see section on supplementary data given at the end of this article).

Therefore using our cohort of cases with multiple tumour foci and matched benign samples, we found that hypermethylation at the *HES5* promoter region was observed across tumour samples from all patients and in all spatially separated tumour foci from the same patient. The homogenous hypermethylation of the *HES5* promoter across genetically heterogeneous tumour cores is consistent with this being an early event in tumourigenesis (Fig. 1c and Supplementary Figure 1m).

### *HES5* promoter methylation is a recurrent event in prostate tumours

**Figure 2:**
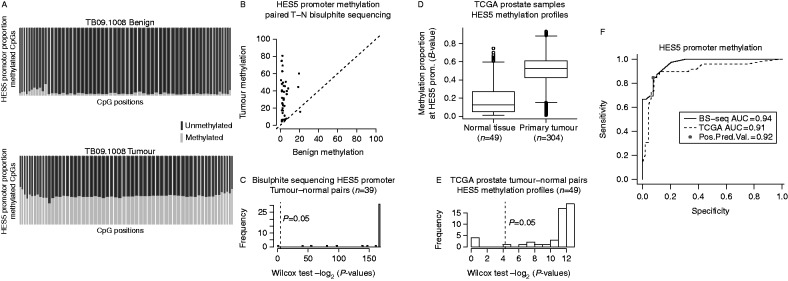
Validation of *HES5* promoter methylation as a common event in two additional independent prostate cancer cohorts. (A) CpG methylation summary of the *HES5* promoter as determined by bisulphite sequencing from a representative tumour–normal pair. Each column represents one CpG assayed (*n*=60), red and blue stacked bars represent the proportion of methylated and unmethylated reads, respectively, at each CpG. Column widths are proportional to sequencing coverage (median=786×). (B) Scatter plot summary of *HES5* promoter methylation for 39 tumour–normal pairs. (C) Histogram summary of significance testing for increased *HES5* promoter methylation in tumour vs normal sample pairs (*n*=39 pairs from panel-C; paired Wilcox rank sum test; −log_2_ *P* values are plotted to visualise distributions). (D) Boxplot summary of *HES5* promoter methylation for 304 tumour and 49 benign prostate samples on Illumina 450k arrays (TCGA data). (E) Histogram summary of significance testing for increased *HES5* promoter methylation in TCGA tumour vs normal sample pairs (*n*=49 pairs from panel-E; paired Wilcox rank sum test; −log_2_ *P* values are plotted to visualise distributions). (F) ROC curve for *HES5* promoter methylation using data from bisulphite sequencing of 39 tumour normal pairs (A, B and C) and methylation array profiling of 49 tumour normal pairs (D and E).

To assess the frequency of *HES5* hypermethylation in prostate cancer, we performed targeted bisulphite sequencing of the *HES5* promoter in a separate cohort of 39 matched tumour and adjacent benign samples. This analysis included 60 CpGs in the *HES5* promoter and gave a median sequencing coverage of 786× (Supplementary Figure 3). This analysis provided a comprehensive view of DNA methylation across the *HES5* gene promoter, in contrast to the four CpGs assessed using methylation arrays and a narrow genomic window in a previous study (Paziewska *et al*. 2014). Benign samples showed hypomethylation across the entire *HES5* promoter, whereas matched tumour samples had consistent hypermethylation across all 60 CpGs assayed (Fig. 2a, b and Supplementary Figure 4, see section on supplementary data given at the end of this article). This pattern of hypomethylation in benign tissue and hypermethylation in tumours was consistent in 38/39 matched tumour normal pairs (97% at *P*<0.05, Wilcox test; Fig. 2c). In the single discordant sample pair, there was increased methylation in the matched benign sample that was maintained in the tumour (median methylation 20.7 and 15.4 respectively; Supplementary Figure 4), consistent with either a pre-transformation change in this single case or tumour contamination of this normal tissue core.

We also assessed *HES5* methylation in an additional prostate cancer patient cohort using publicly available methylation array data (*n*=304 tumours, *n*=49 matched normal samples) (Weinstein *et al*. 2013). In this second validation cohort, we again observed hypermethylation in tumours and hypomethylation in benign samples (42/49 pairs, 86% at *P*<0.05, Wilcox test; Fig. 2d and e). Receiver operating characteristic (ROC) curve analysis for these two geographically distinct validation cohorts run on different platforms revealed high sensitivity and specificity (positive predictive value (PPV)=0.92, area under the curve (AUC)>0.9, Fig. 2f). These results clearly demonstrate that in addition to being an early event in prostate tumourigenesis *HES5* methylation is a highly recurrent event in prostate cancer, suggesting potential as a specific disease marker and an early acquired (or selected) event in prostate tumourigenesis.

### *HES5* is silenced in prostate cancer cells and demethylation restores expression

**Figure 3:**
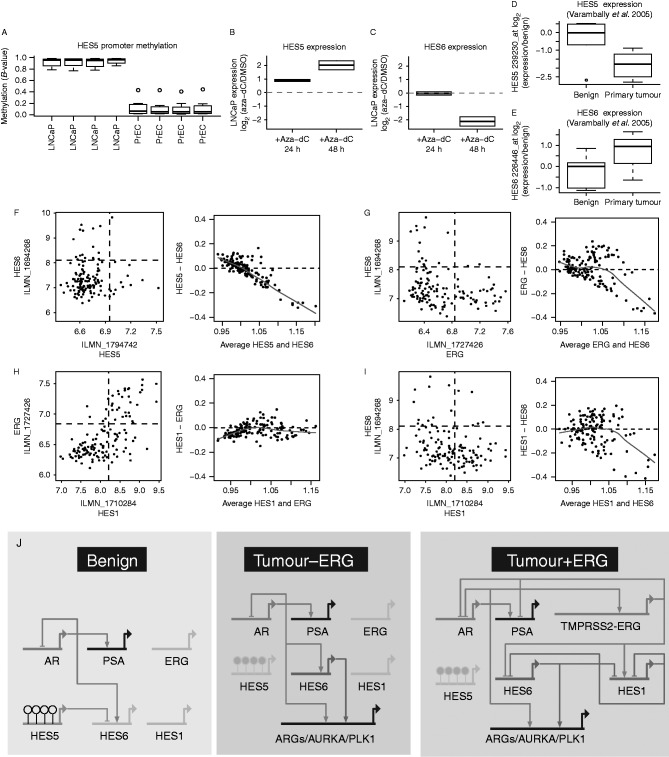
*HES5* expression is repressed by methylation in prostate tumour cells and shows an inverse trend with *HES6* expression. (A) Boxplot showing methylation status of the *HES5* promoter region in LNCaP prostate cancer cells and PrEC benign prostate cells (triplicates from GSE34340 and singletons from GSE40699). (B and C) Expression of (B) *HES5* and (C) *HES6* in LNCaP prostate cancer cells treated with the demethylating agent 5-aza-2′-deoxycytidine (Aza-dC) for 24 and 48 h (GSE25346). Expression presented as log_2_ ratios over control untreated cells. (D and E) Boxplot showing the expression of (D) *HES5* and its known target (E) *HES6* in a separate cohort of prostatic benign and primary tumour tissue (GSE3325). Boxplots depict quartiles, error bars denote 95% CI and data points are shown for values outside 95% CIs. (F, G, H and I) Scatter plots of gene expression from clinical prostate tumours showing the relationship between (F) *HES5* and *HES6*, (G) *HES6* and *ERG*, (H) *HES1* and *ERG*, (I) *HES1* and *HES6* (including samples from the cohort shown in Fig. 2b and c). Plots on the left show pairwise relationships between gene expression, dashed quadrant lines indicates the mid-point of expression values for each gene. Plots on the right show the relationship between the level and difference in expression for each pair of genes (using median centred values for each gene). Divergence from the dashed zero line indicates an inverse relationship, red trend lines depict loess regression. (J) Simple models of the putative expression networks in benign prostate, prostate cancer and ERG-positive prostate cancer involving the *AR*, *HES5*, *HES6*, *ERG* and *HES1*. Genes are depicted by thick horizontal lines, connecting lines depict transcriptional targets of each encoded transcription factor. Connectors with arrowheads depict positively regulated targets, while connectors with flat ends depict repressed targets. Genes shown in grey depict low/no expression in a given condition. On the *HES5* gene open circles depict hypomethylation and filled circles depict hypermethylation. ARGs denotes AR-regulated genes. Model drawn using BioTapestry.

Consistent with observations in human tumours, we found that LNCaP prostate cancer cells exhibit hypermethylation of the *HES5* promoter, in contrast to *HES5* hypomethylation in benign epithelial cells PrEC (Fig. 3a). The expression of *HES5* is low or undetectable in cultured prostate cancer cell lines and is also low in human prostate tumours (Supplementary Figure 5a, c, see section on supplementary data given at the end of this article and Fig. 3d, f), consistent with epigenetic silencing of *HES5* in prostate cancer (Supplementary Figure 5g and h). Treatment of LNCaP cells with the DNA demethylating agent 5-aza-2′-deoxycytidine caused de-repression of the *HES5* gene (Fig. 3b), consistent with active epigenetic silencing of the *HES5* gene in prostate cancer cells.

### *HES5* epigenetic silencing is associated with HES6 expression

*HES5* is known to play a role similar to that of *HES1* in developmental processes (Hatakeyama *et al*. 2004, 2006, Tateya *et al*. 2011), and both are involved in negative feedback loops with HES6 (Fior & Henrique 2005, Jacobsen *et al*. 2008), which antagonises the activity of *HES1* and *HES5* (Bae *et al*. 2000, Salama-Cohen *et al*. 2005). Of note, *HES6* has been recently reported to play an important functional role in prostate cancer enhancing oncogenic signalling through the *AR* (Ramos-Montoya *et al*. 2014). Although a rare *HES6* gene fusion has been reported (Annala *et al*. 2014), no molecular mechanism has been found for the frequent up-regulation of *HES6* in prostate cancer. In prostate cancer cells, de-repression of *HES5* with the demethylating agent 5-aza-2′-deoxycytidine resulted in a delayed downregulation of *HES6* (Fig. 3c), consistent with *HES5* repression of *HES6*. We also observed an inverse relationship between *HES5* and *HES6* expression in a series of primary tumours compared with benign prostate samples, where *HES5* expression decreased and *HES6* expression increased in tumour vs benign prostate samples (Fig. 3d and e). In our cohort of multiple spatially separated tumour samples, we found that *HES5* expression was decreased in tumour cores compared with matched benign tissue and that *HES6* was also increased in some of those tumour cores, consistent with *HES5* silencing in tumourigenesis and additional mechanisms regulating *HES6* expression (Supplementary Figure 5e and f). However, we found no evidence of a correlation between *HES5* methylation and expression in a larger series of tumours (*n*=39), nor between *HES5* and *HES6* expression in this tumour cohort (Fig. 3f). This lack of correlation may at least in part be explained by the low or absent expression of *HES5* in prostate tumour samples (Figs 2 and 3d, f) confounding such correlative analysis. Indeed, we found that *HES5* expression appeared to be low and showed little variation in this series of 39 prostate tumours (Fig. 3f). The few samples that had slightly higher *HES5* expression also had low *HES6* expression (Fig. 3f), which although not compelling alone is consistent with our other data supporting an inverse relationship between *HES5* and *HES6* in addition to highlighting the recurrent silencing of *HES5* in tumourigenesis. There are no successful *HES5* genomic binding data nor chromatin immunoprecipitation grade antibodies for *HES5*; therefore; we could not assess direct binding of *HES5* at the *HES6* gene locus (Yan *et al*. 2013). However, the preferred consensus DNA-binding sequence of *HES5* has been determined experimentally (Yan *et al*. 2013) and we found strong *HES5* consensus sites in and around the *HES6* gene (Supplementary Figure 5i, j and k). Taken together our observations of i) the inverse correlation between *HES5* and *HES6* in cancer cells treated with 5-aza-2′-deoxycytidine, ii) their inverse correlation in tumour-normal comparisons and iii) strong consensus *HES5* binding sites at the *HES6* gene locus suggests that *HES5* may repress *HES6* in prostate epithelial cells. The ubiquitous *HES5* silencing in tumours cells may therefore potentiate (or de-repress) *HES6* expression in prostate tumours.

### *ERG* and *HES6* expression show an inverse relationship

Despite the early and frequent silencing of *HES5* in prostate cancer, we observed variable expression of the *HES5* transcriptional target *HES6* in prostate tumour samples (Fior & Henrique 2005) (Fig. 3f and Supplementary Figure 5f), prompting us to investigate other factors that may regulate *HES6* expression in prostate tumour cells. We found that variations in *HES6* expression showed an inverse relationship with expression of the frequently rearranged *ERG* gene in prostate tumours, highlighted by an inverse correlation (*r*=−0.28) and mutual exclusivity of *HES6* and *ERG* expression (i.e. no samples have both high *ERG* and *HES6* expression, Fig. 3g left panel). This inverse relationship is illustrated clearly by the increasing difference between *ERG* and *HES6* at higher levels of expression (i.e. divergence from zero with increasing expression, Fig. 3g right panel).

### *ERG* and *HES1* expression show a positive correlation

**Figure 4:**
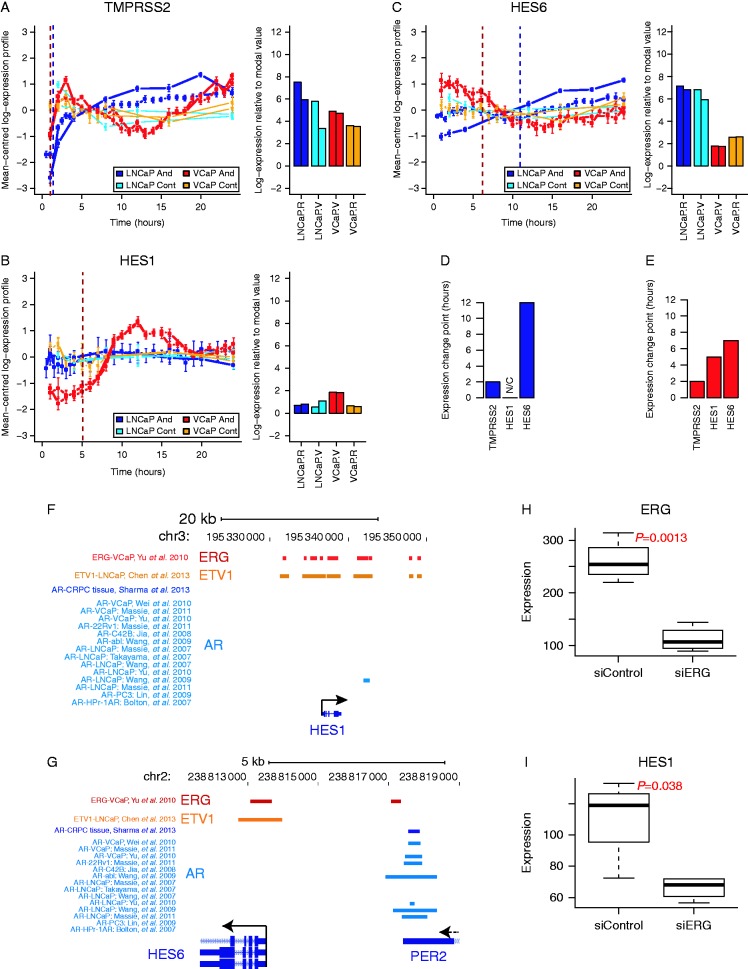
Detailed gene expression time-course analysis, genomic binding profiles and *ERG* knockdown supports an *AR–ERG–HES1–HES6* transcriptional cascade. (A, B and C) Gene expression profiles from androgen stimulation and vehicle control time-course experiments using VCaP (*ERG*-positive) and LNCaP (*ERG*-negative) prostate cancer cells. Panels on the left show the mean centered transcript profiles (as log_2_ ratios/average) and panels on the right show bar plots of the expression levels (log_2_ intensity) for (A) *TMPRSS2*, (B) *HES1* and (C) *HES6*. Error bars depict CI for each time-point measured. Vertical dashed lines correspond to the ‘change-points’ for gene expression in the VCaP (dark red) and LNCaP (dark blue) time-series. (D and E) Bar plots showing the androgen-induced expression ‘change-points’ for each gene from (D) LNCaP and (E) VCaP androgen treatment time-series (values correspond to the dashed lines in panels A, B and C). (F and G) Genomic binding profiles for *ERG*, *ETV1* and the *AR* in prostate cells at the (F) *HES1* and (G) *HES6* gene loci. Genomic binding sites for each transcription factor are depicted by coloured horizontal rectangles. Multiple datasets are included for *AR*-binding profiles using the labelling scheme ‘factor-sample, study’ (i.e. ‘AR-VCaP, Wei *et al*. (2010)’ represents the binding profile of the AR in VCaP cells from the study of Wei *et al*. (2010)). A scale bar is shown at the top together with chromosomal locations and gene locations and orientations are indicated at the bottom of each plot. (H and I) Boxplots showing the expression of (H) *ERG* and (I) *HES1* in VCaP cells under control or *ERG* knockdown conditions (GSE60771). Significance testing was performed using *t*-tests, *P* values annotated on each plot.

In contrast the other major HES6 antagonist HES1 (Bae *et al*. 2000, Hatakeyama *et al*. 2004, 2006, Jacobsen *et al*. 2008) showed a strong positive correlation with *ERG* expression (*r*=0.65; Fig. 3h), suggesting an *ERG*–*HES1*–*HES6* transcriptional network in *ERG*-fusion positive prostate cancer cells (Fig. 3g, h and i). In support of this prediction, we found evidence for extensive *ERG* binding at the *HES1* gene locus (Fig. 4f) and also confirmed the previously reported AR binding sites upstream of the *HES6* gene (Ramos-Montoya *et al*. 2014) by using multiple data sets (Fig. 4g).

### A transcriptional network involving *HES5*, *AR*, *ERG* and *HES6*

Combining our observations of *HES5* silencing in prostate cancer with expression correlations in prostate tissue, DNA binding profiles for *ERG* and the *AR* and published transcriptional links (i.e. between *HES5* and *HES6* (Fior & Henrique 2005), *HES1* and *HES6* (Jacobsen *et al*. 2008), reciprocal HES6 and *HES1/5* negative-feedback (Bae *et al*. 2000, Salama-Cohen *et al*. 2005, Hatakeyama *et al*. 2006) and *AR* and *HES6* (Ramos-Montoya *et al*. 2014)), we constructed models of putative gene expression networks in benign prostate, prostate cancer and prostate cancer harboring *ERG*-rearrangements (Fig. 3j). In this model, we predict that i) *HES5* expression in benign epithelial cells contributes to *HES6* repression and ii) *HES5* promoter methylation and silencing in prostate tumours potentiates *AR* activation of *HES6* to start an oncogenic feed-forward transcriptional signalling network (Fig. 3j). Finally, our model suggests that in tumour cells harbouring an *ERG* gene fusion iii) *AR* activation of the *ERG* fusion gene creates a dynamic negative feedback loop impacting on both the *AR* and *HES6*, creating a more complex transcriptional network (Fig. 3j). Negative feedback loops are common motifs in biological networks and have been shown to increase robustness and speed-up response times of transcriptional circuits (Rosenfeld *et al*. 2002, Shen-Orr *et al*. 2002, Austin *et al*. 2006, Nevozhay *et al*. 2009). Therefore, our model may highlight a previously unknown signalling node in *ERG*-positive tumours that may increase the robustness and response-rates of key pathways in prostate cancer.

### ERG-fusion status affects HES1 and HES6 regulation by the AR

We tested the putative *AR–HES6* and *AR–ERG–HES1–HES6* transcriptional networks in *AR*-positive prostate cancer cells with and without *TMPRSS2–ERG* gene fusions (VCaP and LNCaP, respectively; Fig. 4). Using an androgen stimulation time-course, we were able to both track changes in gene expression and map their dynamics in prostate cancer cells with and without *AR*-regulated *ERG*-fusion expression following *AR* stimulation (Tomlins *et al*. 2005, Massie *et al*. 2011). We observed early up-regulation of the known AR-target gene *TMPRSS2* in both *ERG*-fusion positive and *ERG*-fusion negative cells in response to androgen stimulation (Fig. 4a), while *ERG* induction only occurred in *TMPRSS2–ERG* fusion positive cells (Supplementary Figure 7a, see section on supplementary data given at the end of this article). Consistent with its epigenetic silencing, we found low expression and no change in *HES5* expression in either cell type (Supplementary Figure 7b). *HES1* expression was not significantly changed in *ERG*-fusion negative cells, but showed strong androgen induction in *ERG*-fusion positive cells (Fig. 4b). *HES6* expression was increased in *ERG*-fusion negative cells but was downregulated in *ERG*-fusion positive cells (Fig. 4c). Defining the timing of gene expression changes (‘change-points’) for these genes in *ERG*-fusion positive cells shows the sequence of events: i) *TMPRSS2–ERG* upregulation; ii) *HES1* upregulation; iii) *HES6* downregulation (Fig. 4e). These data show that *HES1* is only induced by androgen signalling in *ERG*-fusion positive cells and that induction precedes *HES6* repression. This transcriptional data are supported by genome-wide binding profiles showing that the AR is recruited to the *HES6* gene locus (Fig. 4g) but not to the *HES1* gene locus in *ERG*-fusion negative cells (Fig. 4f). However, in *ERG*-fusion positive cells, *ERG* binding is widespread at the *HES1* locus (Fig. 4f), consistent with direct *ERG* regulation of the *HES1* gene.

### HES1 expression is dependent on ERG

To test this further, we looked at the expression of *HES1* following *ERG* knockdown in VCaP cells (Mounir *et al*. 2014) (Fig. 4h and Supplementary Figure 6b, c, see section on supplementary data given at the end of this article) and found that *HES1* expression was dependent on the expression of *ERG* (Fig. 4i and Supplementary Figure 6d, e), further supporting our model. In addition to the timing of expression changes in response to androgen stimulation, these data support an *AR–ERG–HES1–HES6* transcriptional network in *ERG*-fusion positive prostate cancer cells. While in *ERG*-fusion negative cells, a simpler *AR–HES6* network seems to occur. In each case, these transcriptional networks may have been preceded (and potentiated) by *HES5* epigenetic silencing in early tumourigenesis.

## Discussion

Our data are consistent with an early role in prostate tumourigenesis for promoter-wide hypermethylation of *HES5*, supported by the very high frequency of this epigenetic change and our observation that this was a common alteration in a series of multi-focal tumours. While the functional role of *HES5* methylation in prostate tumourigenesis is yet to be determined, we found that demethylation resulted in downregulation of the *HES5*-target gene *HES6*, which has recently been shown to drive progression in prostate cancer via the androgen receptor (Ramos-Montoya *et al*. 2014). Therefore, we speculate that one potential effector mechanism of *HES5* silencing could be de-repression of *HES6* that in turn enhances AR regulation of key oncogenic targets, contributing to transformation and/or priming cells for subsequent acquisition of aggressive phenotypes. In addition, *HES5* has established roles in tissue patterning during development (Hatakeyama *et al*. 2004, Tateya *et al*. 2011), with *HES5*-null cells promoting an imbalance in intestinal and neural stem cell fate choices resulting from defective NOTCH signalling (Sancho *et al*. 2013). Intriguingly defective NOTCH signalling has recently been shown to drive clonal expansions of P53 mutant cells (Alcolea *et al*. 2014), raising the possibility that *HES5* silencing early in prostate tumourogenesis might drive clonal expansions and contribute to the ‘field effect’ observed in prostate tumours (Bostwick *et al*. 1998, Hanson *et al*. 2006, Mehrotra *et al*. 2008). However, these and other downstream consequences of the early and common epigenetic silencing of *HES5* will require careful dissection in future studies.

It is intriguing that this *HES5–HES6*/*AR–HES6* transcriptional network is affected by *TMPRSS2–ERG* gene fusion status. While the functional consequences of this remain to be explored, the implication of both *AR* and *ERG* oncogenenic signalling axes provides further weight for the importance of the *HES* transcriptional network in prostate cancer. Future studies will need to include overexpression of *HES5* in prostate cancer cells to establish the direct consequences on *HES6* and *AR* signalling, as well as the phenotypic consequences of bypassing *HES5* silencing. In addition, depletion of *HES5* in 5-aza-2′-deoxycytidine-treated prostate cancer cells (both *ERG*-positive and *ERG*-negative) will allow an assessment of de-repression of the endogenous *HES5* locus on gene expression and cellular phenotypes. Finally, future studies should also address the mechanisms upstream of *HES5* silencing, the high frequency of which would be consistent with either a strong-selective pressure or a targeted silencing of *HES5*, for example via loss of GCM as described in neural stem cells (Hitoshi *et al*. 2011).

This report highlights *HES5* silencing as an early and frequent event in prostate tumourigenesis that may serve as a useful biomarker or as a starting point for preventive medicine or targeted intervention strategies.

## Supplementary data

This is linked to the online version of the paper at http://dx.doi.org/10.1530/ERC-14-0454.

## Author contribution statement

C E Massie carried out analysis, directed the project and wrote the manuscript; I Spiteri designed and carried out bisulphite sequencing experiments; H Ross-Adams prepared samples for bisulphite sequencing and tumour gene expression data; H Luxton, J Kay and H C Whitaker co-designed and provided all samples for the initial methylation profiling, expression and validation experiments; M J Dunning, A D Lamb and A Ramos-Montoya provided tumour gene expression data; A Y Warren carried out systematic pathology of prostatectomy samples and identified tumour and benign cores for sampling; S Tavaré designed the study and drafted the manuscript; D S Brewer, C S Cooper, R Eeles and the ICGC Working Group provided 3D prostate reconstructions, contributed to study design and drafted the manuscript; D E Neal co-designed the study and drafted the manuscript; A G Lynch co-designed the study, carried out analysis, directed the project and co-authored the manuscript.

## Supplementary Material

Supplementary Figure

Supplementary Data

Supplementary Data

## References

[bib1] Alcolea MP, Greulich P, Wabik A, Frede J, Simons BD, Jones PH (2014). Differentiation imbalance in single oesophageal progenitor cells causes clonal immortalization and field change. Nature Cell Biology.

[bib2] Annala M, Kivinummi K, Leinonen K, Tuominen J, Zhang W, Visakorpi T, Nykter M (2014). DOT1L-HES6 fusion drives androgen independent growth in prostate cancer. EMBO Molecular Medicine.

[bib3] Aryee MJ, Jaffe AE, Corrada-Bravo H, Ladd-Acosta C, Feinberg AP, Hansen KD, Irizarry RA (2014). Minfi: a flexible and comprehensive Bioconductor package for the analysis of Infinium DNA methylation microarrays. Bioinformatics.

[bib4] Austin DW, Allen MS, McCollum JM, Dar RD, Wilgus JR, Sayler GS, Samatova NF, Cox CD, Simpson ML (2006). Gene network shaping of inherent noise spectra. Nature.

[bib5] Baca SC, Prandi D, Lawrence MS, Mosquera JM, Romanel A, Drier Y, Park K, Kitabayashi N, MacDonald TY, Ghandi M (2013). Punctuated evolution of prostate cancer genomes. Cell.

[bib6] Bae S, Bessho Y, Hojo M, Kageyama R (2000). The bHLH gene Hes6, an inhibitor of Hes1, promotes neuronal differentiation. Development.

[bib7] Barbieri CE, Baca SC, Lawrence MS, Demichelis F, Blattner M, Theurillat JP, White TA, Stojanov P, Van Allen E, Stransky N (2012). Exome sequencing identifies recurrent SPOP, FOXA1 and MED12 mutations in prostate cancer. Nature Genetics.

[bib8] Barry M, Perner S, Demichelis F, Rubin MA (2007). TMPRSS2-ERG fusion heterogeneity in multifocal prostate cancer: clinical and biologic implications. Urology.

[bib9] Berger MF, Lawrence MS, Demichelis F, Drier Y, Cibulskis K, Sivachenko AY, Sboner A, Esgueva R, Pflueger D, Sougnez C (2011). The genomic complexity of primary human prostate cancer. Nature.

[bib10] Biankin AV, Waddell N, Kassahn KS, Gingras MC, Muthuswamy LB, Johns AL, Miller DK, Wilson PJ, Patch AM, Wu J (2012). Pancreatic cancer genomes reveal aberrations in axon guidance pathway genes. Nature.

[bib80] Bolton EC, So AY, Chaivorapol C, Haqq CM, Li H, Yamamoto KR (2007). Cell- and gene-specific regulation of primary target genes by the androgen receptor. Genes and Development.

[bib11] Bostwick DG, Shan A, Qian J, Darson M, Maihle NJ, Jenkins RB, Cheng L (1998). Independent origin of multiple foci of prostatic intraepithelial neoplasia: comparison with matched foci of prostate carcinoma. Cancer.

[bib12] Caini S, Gandini S, Dudas M, Bremer V, Severi E, Gherasim A (2014). Sexually transmitted infections and prostate cancer risk: a systematic review and meta-analysis. Cancer Epidemiology.

[bib13] Cairns JM, Dunning MJ, Ritchie ME, Russell R, Lynch AG (2008). BASH: a tool for managing BeadArray spatial artefacts. Bioinformatics.

[bib14] Cancer Genome Atlas Network (2012). Comprehensive molecular characterization of human colon and rectal cancer. Nature.

[bib15] Cancer Genome Atlas Research Network (2011). Integrated genomic analyses of ovarian carcinoma. Nature.

[bib16] Carver BS, Tran J, Gopalan A, Chen Z, Shaikh S, Carracedo A, Alimonti A, Nardella C, Varmeh S, Scardino PT (2009). Aberrant ERG expression cooperates with loss of PTEN to promote cancer progression in the prostate. Nature Genetics.

[bib17] Chen Y, Chi P, Rockowitz S, Iaquinta PJ, Shamu T, Shukla S, Gao D, Sirota I, Carver BS, Wongvipat J (2013). ETS factors reprogram the androgen receptor cistrome and prime prostate tumorigenesis in response to PTEN loss. Nature Medicine.

[bib18] Davis S, Meltzer PS (2007). GEOquery: a bridge between the Gene Expression Omnibus (GEO) and BioConductor. Bioinformatics.

[bib19] Dunning MJ, Smith ML, Ritchie ME, Tavare S (2007). beadarray: R classes and methods for Illumina bead-based data. Bioinformatics.

[bib20] Eeles RA, Olama AA, Benlloch S, Saunders EJ, Leongamornlert DA, Tymrakiewicz M, Ghoussaini M, Luccarini C, Dennis J, Jugurnauth-Little S (2013). Identification of 23 new prostate cancer susceptibility loci using the iCOGS custom genotyping array. Nature Genetics.

[bib21] Elkahwaji JE, Hauke RJ, Brawner CM (2009). Chronic bacterial inflammation induces prostatic intraepithelial neoplasia in mouse prostate. British Journal of Cancer.

[bib22] Fior R, Henrique D (2005). A novel hes5/hes6 circuitry of negative regulation controls Notch activity during neurogenesis. Developmental Biology.

[bib23] Freedland SJ (2005). Obesity and prostate cancer: a growing problem. Clinical Cancer Research.

[bib24] Garcia AJ, Ruscetti M, Arenzana TL, Tran LM, Bianci-Frias D, Sybert E, Priceman SJ, Wu L, Nelson PS, Smale ST (2014). Pten null prostate epithelium promotes localized myeloid-derived suppressor cell expansion and immune suppression during tumor initiation and progression. Molecular and Cellular Biology.

[bib25] Grasso CS, Wu YM, Robinson DR, Cao X, Dhanasekaran SM, Khan AP, Quist MJ, Jing X, Lonigro RJ, Brenner JC (2012). The mutational landscape of lethal castration-resistant prostate cancer. Nature.

[bib26] Hanson JA, Gillespie JW, Grover A, Tangrea MA, Chuaqui RF, Emmert-Buck MR, Tangrea JA, Libutti SK, Linehan WM, Woodson KG (2006). Gene promoter methylation in prostate tumor-associated stromal cells. Journal of the National Cancer Institute.

[bib27] Hatakeyama J, Bessho Y, Katoh K, Ookawara S, Fujioka M, Guillemot F, Kageyama R (2004). Hes genes regulate size, shape and histogenesis of the nervous system by control of the timing of neural stem cell differentiation. Development.

[bib28] Hatakeyama J, Sakamoto S, Kageyama R (2006). Hes1 and Hes5 regulate the development of the cranial and spinal nerve systems. Developmental Neuroscience.

[bib29] Heaphy CM, Yoon GS, Peskoe SB, Joshu CE, Lee TK, Giovannucci E, Mucci LA, Kenfield SA, Stampfer MJ, Hicks JL (2013). Prostate cancer cell telomere length variability and stromal cell telomere length as prognostic markers for metastasis and death. Cancer Discovery.

[bib30] Hitoshi S, Ishino Y, Kumar A, Jasmine S, Tanaka KF, Kondo T, Kato S, Hosoya T, Hotta Y, Ikenaka K (2011). Mammalian Gcm genes induce Hes5 expression by active DNA demethylation and induce neural stem cells. Nature Neuroscience.

[bib31] Jacobsen KX, Vanderluit JL, Slack RS, Albert PR (2008). HES1 regulates 5-HT1A receptor gene transcription at a functional polymorphism: essential role in developmental expression. Molecular and Cellular Neurosciences.

[bib74] Jia L, Berman BP, Jariwala U, Yan X, Cogan JP, Walters A, Chen T, Buchanan G, Frenkel B, Coetzee GA (2008). Genomic androgen receptor-occupied regions with different functions, defined by histone acetylation, coregulators and transcriptional capacity. PLoS ONE.

[bib32] Kalaany NY, Sabatini DM (2009). Tumours with PI3K activation are resistant to dietary restriction. Nature.

[bib33] Kanwal R, Pandey M, Bhaskaran N, Maclennan GT, Fu P, Ponsky LE, Gupta S (2014). Protection against oxidative DNA damage and stress in human prostate by glutathione S-transferase P1. Molecular Carcinogenesis.

[bib34] Kote-Jarai Z, Olama AA, Giles GG, Severi G, Schleutker J, Weischer M, Campa D, Riboli E, Key T, Gronberg H (2011). Seven prostate cancer susceptibility loci identified by a multi-stage genome-wide association study. Nature Genetics.

[bib35] Krueger F, Andrews SR (2011). Bismark: a flexible aligner and methylation caller for Bisulfite-Seq applications. Bioinformatics.

[bib36] Kwon OJ, Zhang L, Ittmann MM, Xin L (2014). Prostatic inflammation enhances basal-to-luminal differentiation and accelerates initiation of prostate cancer with a basal cell origin. PNAS.

[bib37] Lee WH, Morton RA, Epstein JI, Brooks JD, Campbell PA, Bova GS, Hsieh WS, Isaacs WB, Nelson WG (1994). Cytidine methylation of regulatory sequences near the pi-class glutathione S-transferase gene accompanies human prostatic carcinogenesis. PNAS.

[bib79] Lin B, Wang J, Hong X, Yan X, Hwang D, Cho JH, Yi D, Utleg AG, Fang X, Schones DE (2009). Integrated expression profiling and ChIP-seq analyses of the growth inhibition response program of the androgen receptor. PLoS ONE.

[bib38] Lindberg J, Klevebring D, Liu W, Neiman M, Xu J, Wiklund P, Wiklund F, Mills IG, Egevad L, Gronberg H (2013). Exome sequencing of prostate cancer supports the hypothesis of independent tumour origins. European Urology.

[bib39] Longabaugh WJ (2012). BioTapestry: a tool to visualize the dynamic properties of gene regulatory networks. Methods in Molecular Biology.

[bib40] Marabita F, Almgren M, Lindholm ME, Ruhrmann S, Fagerstrom-Billai F, Jagodic M, Sundberg CJ, Ekstrom TJ, Teschendorff AE, Tegner J (2013). An evaluation of analysis pipelines for DNA methylation profiling using the Illumina HumanMethylation450 BeadChip platform. Epigenetics.

[bib77] Massie CE, Adryan B, Barbosa-Morais NL, Lynch AG, Tran MG, Neal DE, Mills IG (2007). New androgen receptor genomic targets show an interaction with the ETS1 transcription factor. EMBO Reproduction.

[bib41] Massie CE, Lynch A, Ramos-Montoya A, Boren J, Stark R, Fazli L, Warren A, Scott H, Madhu B, Sharma N (2011). The androgen receptor fuels prostate cancer by regulating central metabolism and biosynthesis. EMBO Journal.

[bib42] Mehrotra J, Varde S, Wang H, Chiu H, Vargo J, Gray K, Nagle RB, Neri JR, Mazumder A (2008). Quantitative, spatial resolution of the epigenetic field effect in prostate cancer. Prostate.

[bib43] Mertz KD, Horcic M, Hailemariam S, D'Antonio A, Dirnhofer S, Hartmann A, Agaimy A, Eppenberger-Castori S, Obermann E, Cathomas G (2013). Heterogeneity of ERG expression in core needle biopsies of patients with early prostate cancer. Human Pathology.

[bib44] Minner S, Gartner M, Freudenthaler F, Bauer M, Kluth M, Salomon G, Heinzer H, Graefen M, Bokemeyer C, Simon R (2013). Marked heterogeneity of ERG expression in large primary prostate cancers. Modern Pathology.

[bib45] Mounir Z, Lin F, Lin VG, Korn JM, Yu Y, Valdez R, Aina OH, Buchwalter G, Jaffe AB, Korpal M (2014). TMPRSS2:ERG blocks neuroendocrine and luminal cell differentiation to maintain prostate cancer proliferation. Oncogene.

[bib46] Nevozhay D, Adams RM, Murphy KF, Josic K, Balazsi G (2009). Negative autoregulation linearizes the dose–response and suppresses the heterogeneity of gene expression. PNAS.

[bib47] Nik-Zainal S, Van Loo P, Wedge DC, Alexandrov LB, Greenman CD, Lau KW, Raine K, Jones D, Marshall J, Ramakrishna M (2012). The life history of 21 breast cancers. Cell.

[bib48] Paziewska A, Dabrowska M, Goryca K, Antoniewicz A, Dobruch J, Mikula M, Jarosz D, Zapala L, Borowka A, Ostrowski J (2014). DNA methylation status is more reliable than gene expression at detecting cancer in prostate biopsy. British Journal of Cancer.

[bib49] Perner S, Demichelis F, Beroukhim R, Schmidt FH, Mosquera JM, Setlur S, Tchinda J, Tomlins SA, Hofer MD, Pienta KG (2006). TMPRSS2:ERG fusion-associated deletions provide insight into the heterogeneity of prostate cancer. Cancer Research.

[bib50] Pollock RF, Adryan B (2008). BioSAVE: display of scored annotation within a sequence context. BMC Bioinformatics.

[bib51] Ramos-Montoya A, Lamb AD, Russell R, Carroll T, Jurmeister S, Galeano-Dalmau N, Massie CE, Boren J, Bon H, Theodorou V (2014). HES6 drives a critical AR transcriptional programme to induce castration-resistant prostate cancer through activation of an E2F1-mediated cell cycle network. EMBO Molecular Medicine.

[bib52] R-Core-Team (2014). R: A Language and Environment for Statistical Computing.

[bib53] Rosenfeld N, Elowitz MB, Alon U (2002). Negative autoregulation speeds the response times of transcription networks. Journal of Molecular Biology.

[bib54] Salama-Cohen P, Arevalo MA, Meier J, Grantyn R, Rodriguez-Tebar A (2005). NGF controls dendrite development in hippocampal neurons by binding to p75NTR and modulating the cellular targets of Notch. Molecular Biology of the Cell.

[bib55] Sancho R, Blake SM, Tendeng C, Clurman BE, Lewis J, Behrens A (2013). Fbw7 repression by hes5 creates a feedback loop that modulates Notch-mediated intestinal and neural stem cell fate decisions. PLoS Biology.

[bib72] Sharma NL, Massie CE, Ramos-Montoya A, Zecchini V, Scott HE, Lamb AD, MacArthur S, Stark R, Warren AY, Mills IG (2013). The androgen receptor induces a distinct transcriptional program in castration-resistant prostate cancer in man. Cancer Cell.

[bib56] Shen-Orr SS, Milo R, Mangan S, Alon U (2002). Network motifs in the transcriptional regulation network of *Escherichia coli*. Nature Genetics.

[bib57] Sommerfeld HJ, Meeker AK, Piatyszek MA, Bova GS, Shay JW, Coffey DS (1996). Telomerase activity: a prevalent marker of malignant human prostate tissue. Cancer Research.

[bib58] Statham AL, Robinson MD, Song JZ, Coolen MW, Stirzaker C, Clark SJ (2012). Bisulfite sequencing of chromatin immunoprecipitated DNA (BisChIP-seq) directly informs methylation status of histone-modified DNA. Genome Research.

[bib78] Takayama K, Kaneshiro K, Tsutsumi S, Horie-Inoue K, Ikeda K, Urano T, Ijichi N, Ouchi Y, Shirahige K, Aburatani H (2007). Identification of novel androgen response genes in prostate cancer cells by coupling chromatin immunoprecipitation and genomic microarray analysis. Oncogene.

[bib59] Tarpey PS, Behjati S, Cooke SL, Van Loo P, Wedge DC, Pillay N, Marshall J, O'Meara S, Davies H, Nik-Zainal S (2013). Frequent mutation of the major cartilage collagen gene COL2A1 in chondrosarcoma. Nature Genetics.

[bib60] Tateya T, Imayoshi I, Tateya I, Ito J, Kageyama R (2011). Cooperative functions of Hes/Hey genes in auditory hair cell and supporting cell development. Developmental Biology.

[bib61] Tomlins SA, Rhodes DR, Perner S, Dhanasekaran SM, Mehra R, Sun XW, Varambally S, Cao X, Tchinda J, Kuefer R (2005). Recurrent fusion of TMPRSS2 and ETS transcription factor genes in prostate cancer. Science.

[bib62] Turatsinze JV, Thomas-Chollier M, Defrance M, van Helden J (2008). Using RSAT to scan genome sequences for transcription factor binding sites and cis-regulatory modules. Nature Protocols.

[bib63] Varley KE, Gertz J, Bowling KM, Parker SL, Reddy TE, Pauli-Behn F, Cross MK, Williams BA, Stamatoyannopoulos JA, Crawford GE (2013). Dynamic DNA methylation across diverse human cell lines and tissues. Genome Research.

[bib75] Wang Q, Li W, Liu XS, Carroll JS, Jänne OA, Keeton EK, Chinnaiyan AM, Pienta KJ, Brown M (2007). A hierarchical network of transcription factors governs androgen receptor-dependent prostate cancer growth. Molecular Cell.

[bib76] Wang Q, Li W, Zhang Y, Yuan X, Xu K, Yu J, Chen Z, Beroukhim R, Wang H, Lupien M (2009). Androgen receptor regulates a distinct transcription program in androgen-independent prostate cancer. Cell.

[bib64] Warren AY, Whitaker HC, Haynes B, Sangan T, McDuffus LA, Kay JD, Neal DE (2013). Method for sampling tissue for research which preserves pathological data in radical prostatectomy. Prostate.

[bib65] Weaver JM, Ross-Innes CS, Shannon N, Lynch AG, Forshew T, Barbera M, Murtaza M, Ong CA, Lao-Sirieix P, Dunning MJ (2014). Ordering of mutations in preinvasive disease stages of esophageal carcinogenesis. Nature Genetics.

[bib73] Wei GH, Badis G, Berger MF, Kivioja T, Palin K, Enge M, Bonke M, Jolma A, Varjosalo M, Gehrke AR (2010). Genome-wide analysis of ETS-family DNA-binding *in vitro* and *in vivo*. EMBO Journal.

[bib66] Weinstein JN, Collisson EA, Mills GB, Shaw KR, Ozenberger BA, Ellrott K, Shmulevich I, Sander C, Stuart JM (2013). The Cancer Genome Atlas Pan-Cancer analysis project. Nature Genetics.

[bib67] Weischenfeldt J, Simon R, Feuerbach L, Schlangen K, Weichenhan D, Minner S, Wuttig D, Warnatz HJ, Stehr H, Rausch T (2013). Integrative genomic analyses reveal an androgen-driven somatic alteration landscape in early-onset prostate cancer. Cancer Cell.

[bib68] Yan J, Enge M, Whitington T, Dave K, Liu J, Sur I, Schmierer B, Jolma A, Kivioja T, Taipale M (2013). Transcription factor binding in human cells occurs in dense clusters formed around cohesin anchor sites. Cell.

[bib71] Yu J, Yu J, Mani RS, Cao Q, Brenner CJ, Cao X, Wang X, Wu L, Li J, Hu M (2010). An integrated network of androgen receptor, polycomb, and TMPRSS2-ERG gene fusions in prostate cancer progression. Cancer Cell.

