# Supplementary material for: HES5 silencing is an early and recurrent change in prostate tumourigenesis
Source: Endocr Relat Cancer. 2015 Jan 5;22(2):131–44. doi: 10.1530/ERC-14-0454 (PMC4335379; doi:10.1530/ERC-14-0454)
Supplement: Supplementary Figure [file supp_22_2_131__index.html]

HES5 silencing is an early and recurrent change in prostate tumourigenesis — HES5 silencing is an early and recurrent change in prostate tumourigenesis — HES5 silenced early in prostate tumourigenesis — Supplementary Figure 

# *HES5* silencing is an early and recurrent change in prostate tumourigenesis

## Supplementary Figure

**Files in this Data Supplement:**

- Supplementary Figure 1 - (PDF 732 KB)
- Supplementary Figure 2 - (PDF 571 KB)
- Supplementary Figure 3 - (PDF 330 KB)
- Supplementary Figure 4 - (PDF 1,155 KB)
- Supplementary Figure 5 - (PDF 1,098 KB)
- Supplementary Figure 6 - (PDF 210 KB)
- Supplementary Figure 7 - (PDF 437 KB)
