# Supplementary figures and images for: HES5 silencing is an early and recurrent change in prostate tumourigenesis
Source: Endocr Relat Cancer. 2015 Jan 5;22(2):131–44. doi: 10.1530/ERC-14-0454 (PMC4335379; doi:10.1530/ERC-14-0454)

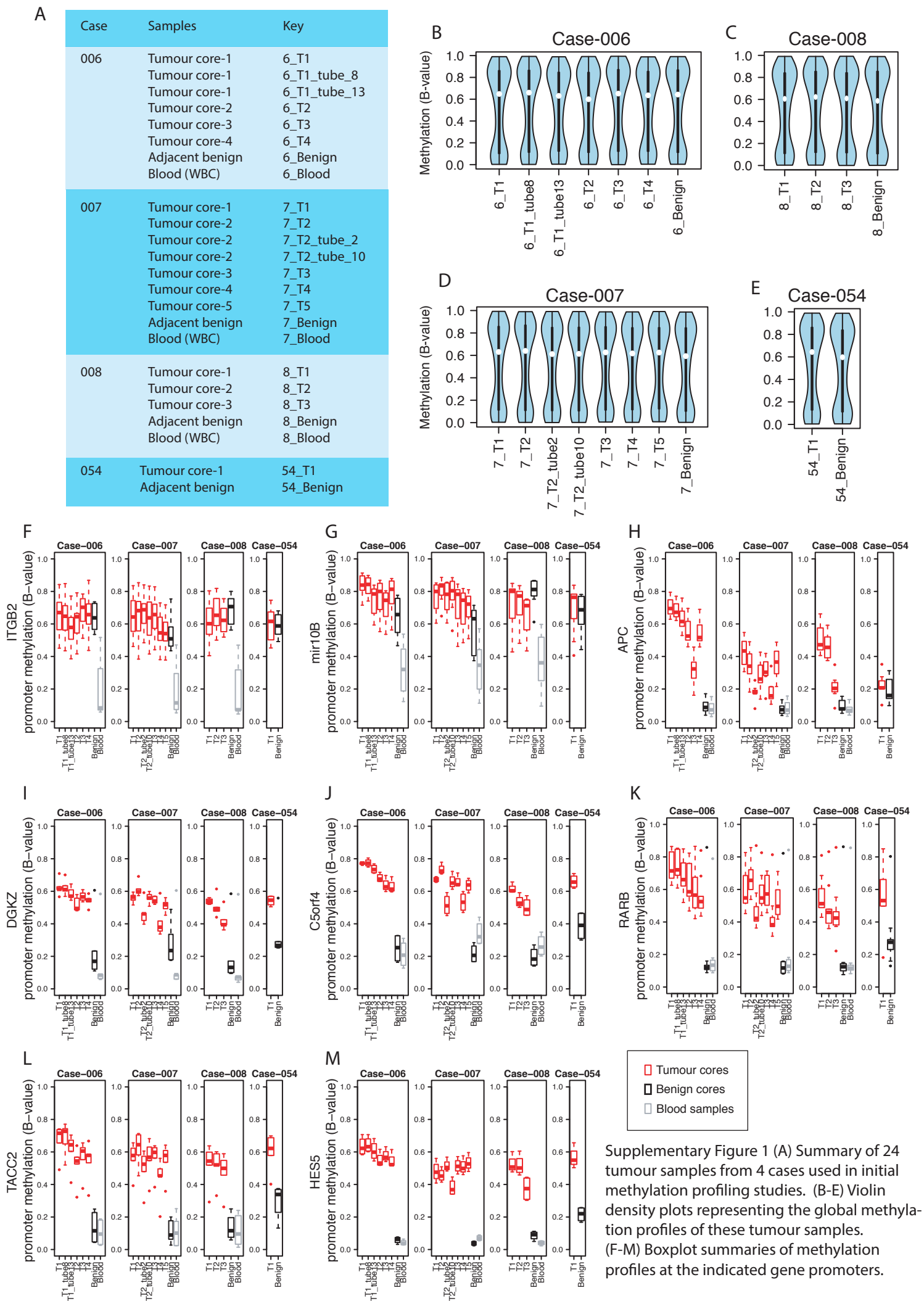

Supplement: Supplementary Figure [file supp_ERC-14-0454_Supplementary_figure_1.pdf]
