# Supplementary material for: HES5 silencing is an early and recurrent change in prostate tumourigenesis
Source: Endocr Relat Cancer. 2015 Jan 5;22(2):131–44. doi: 10.1530/ERC-14-0454 (PMC4335379; doi:10.1530/ERC-14-0454)
Supplement: Supplementary Figure [file supp_ERC-14-0454_Supplementary_figure_2.pdf]

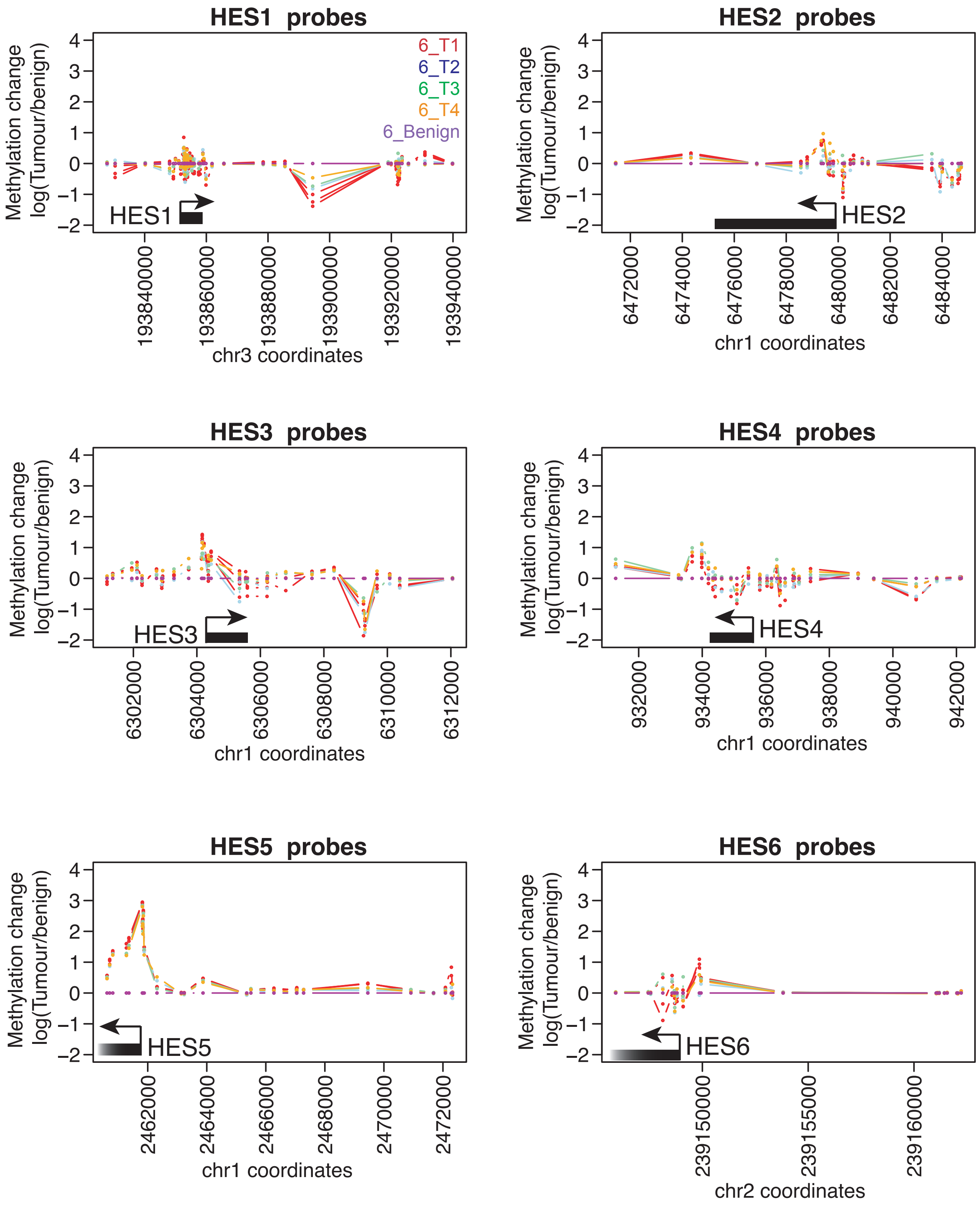

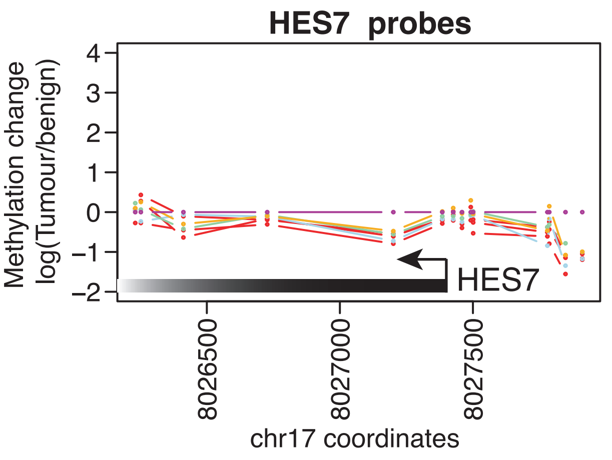

Supplementary Figure 2 Methylation profiles of HES gene loci from human prostate tumours. Plots show the log ratios of methylation in tumour over benign samples for all probes on the hm450k array with an annotation for HES genes. Methylation ratios are plotted for each probe and for each sample against the genomic location of thier target sequence. HES gene locations are indicated below plots for each gene, arrows indicate transcript orientation.
