# Supplementary material for: HES5 silencing is an early and recurrent change in prostate tumourigenesis
Source: Endocr Relat Cancer. 2015 Jan 5;22(2):131–44. doi: 10.1530/ERC-14-0454 (PMC4335379; doi:10.1530/ERC-14-0454)
Supplement: Supplementary Figure [file supp_ERC-14-0454_Supplementary_figure_3.pdf]

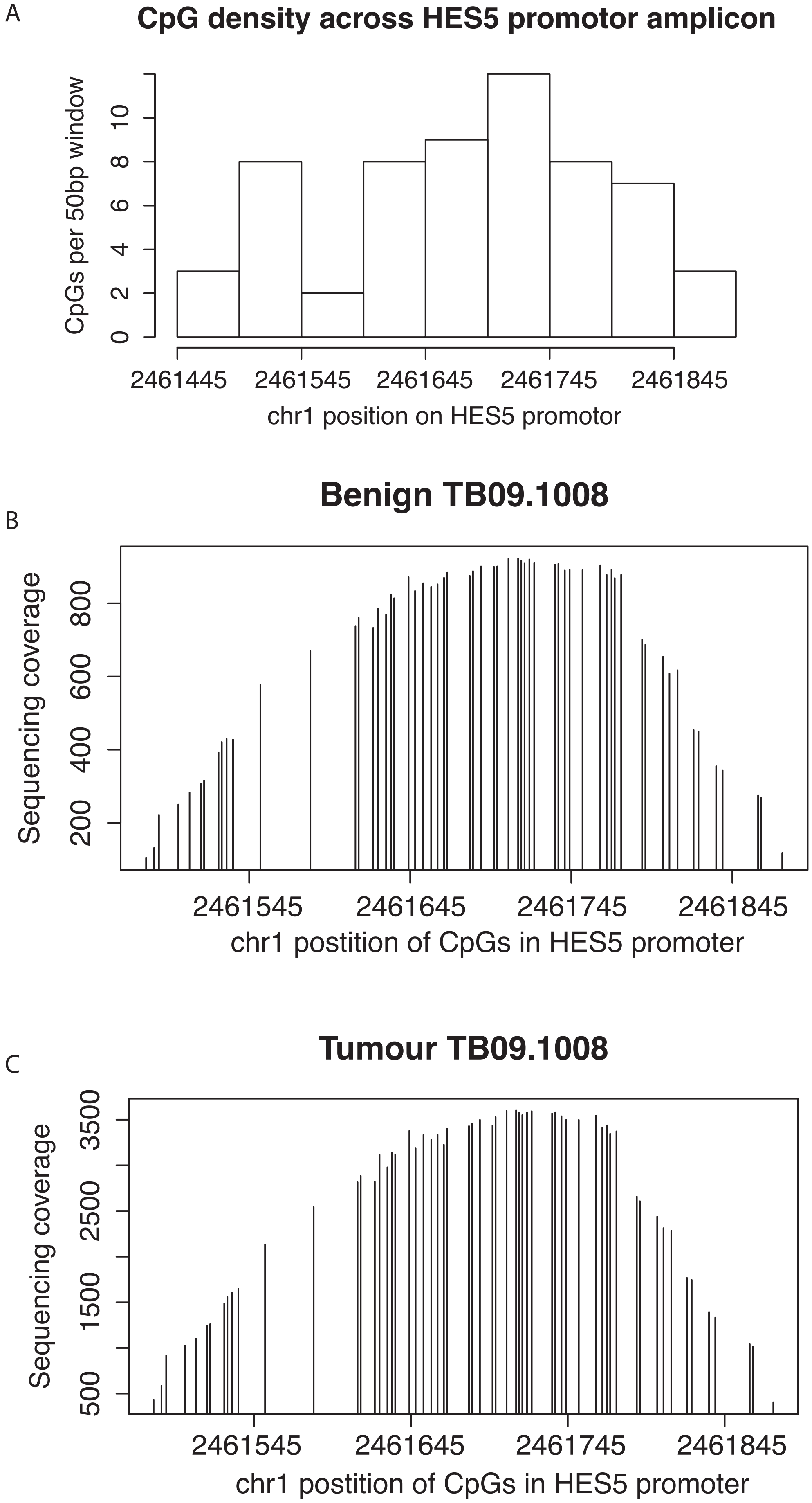

Supplementary Figure 3 Summary of amplicon bisulfite sequencing of the HES5 promoter region in 39 tumour normal pairs. (A) CpG density across the 441bp HES5 promoter amplicon. (B-C) Sequencing coverage for each CpG assayed across the HES5 promoter amplicon for representative (B) benign and (C) tumour samples.
