# Supplementary material for: HES5 silencing is an early and recurrent change in prostate tumourigenesis
Source: Endocr Relat Cancer. 2015 Jan 5;22(2):131–44. doi: 10.1530/ERC-14-0454 (PMC4335379; doi:10.1530/ERC-14-0454)
Supplement: Supplementary Figure [file supp_ERC-14-0454_Supplementary_figure_4.pdf]

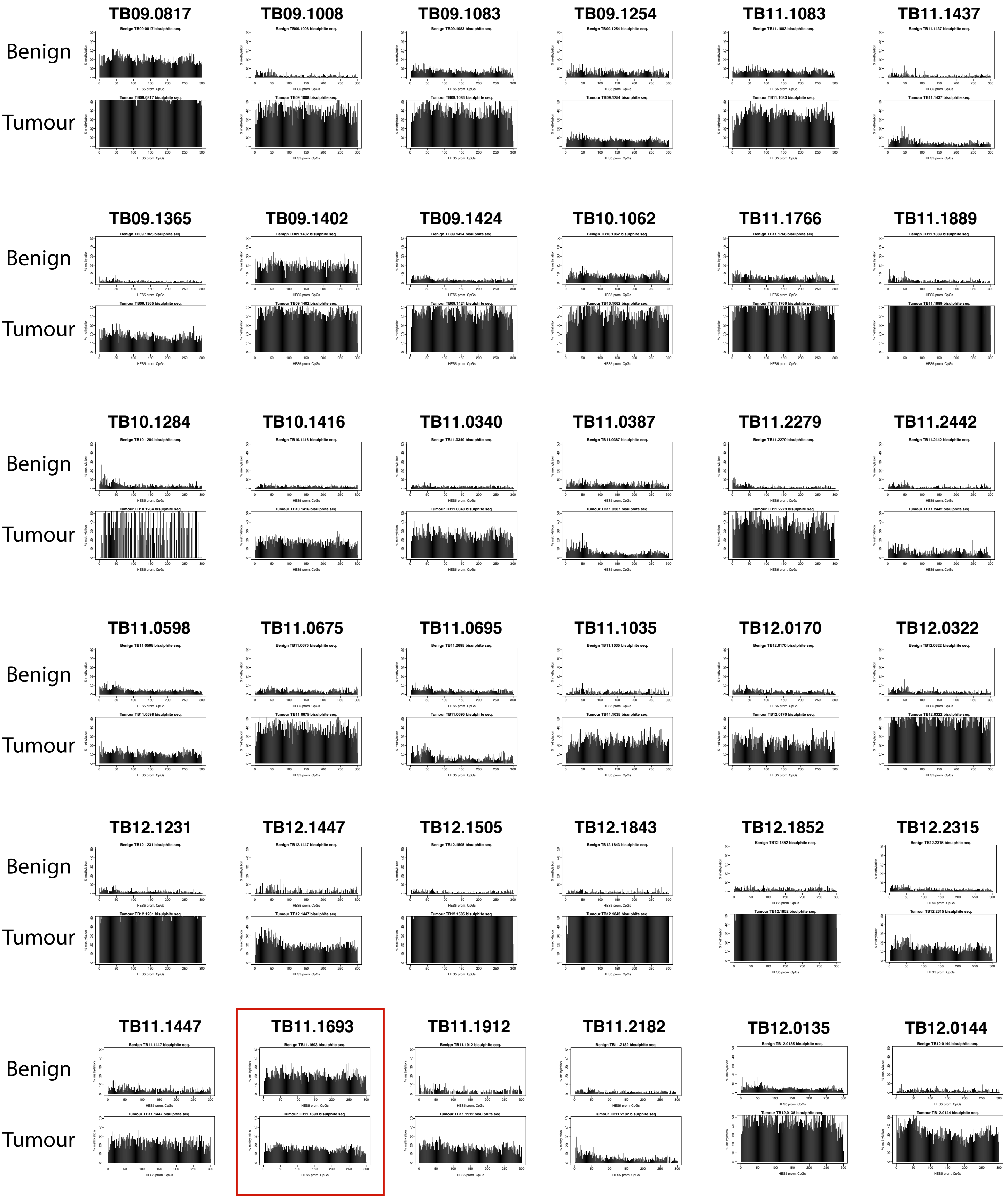

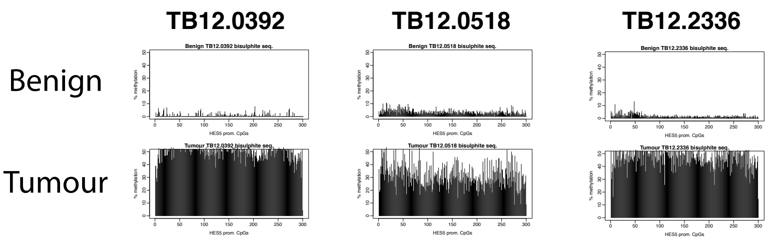

Supplementary Figure 4 Bisulfite sequencing of the HES5 promoter in 39 matched Tumour-Normal pairs. Each pair of plots shows benign (above) and tumour (below) for a single patient. Methylation percentage (y-axis, fixed 0-50% scale) is shown for each of 60 CpGs assayed (x-axis). Red box high-lights the single sample pair without increased methylation in tumour vs benign.
