# Supplementary material for: HES5 silencing is an early and recurrent change in prostate tumourigenesis
Source: Endocr Relat Cancer. 2015 Jan 5;22(2):131–44. doi: 10.1530/ERC-14-0454 (PMC4335379; doi:10.1530/ERC-14-0454)
Supplement: Supplementary Figure [file supp_ERC-14-0454_Supplementary_figure_5.pdf]

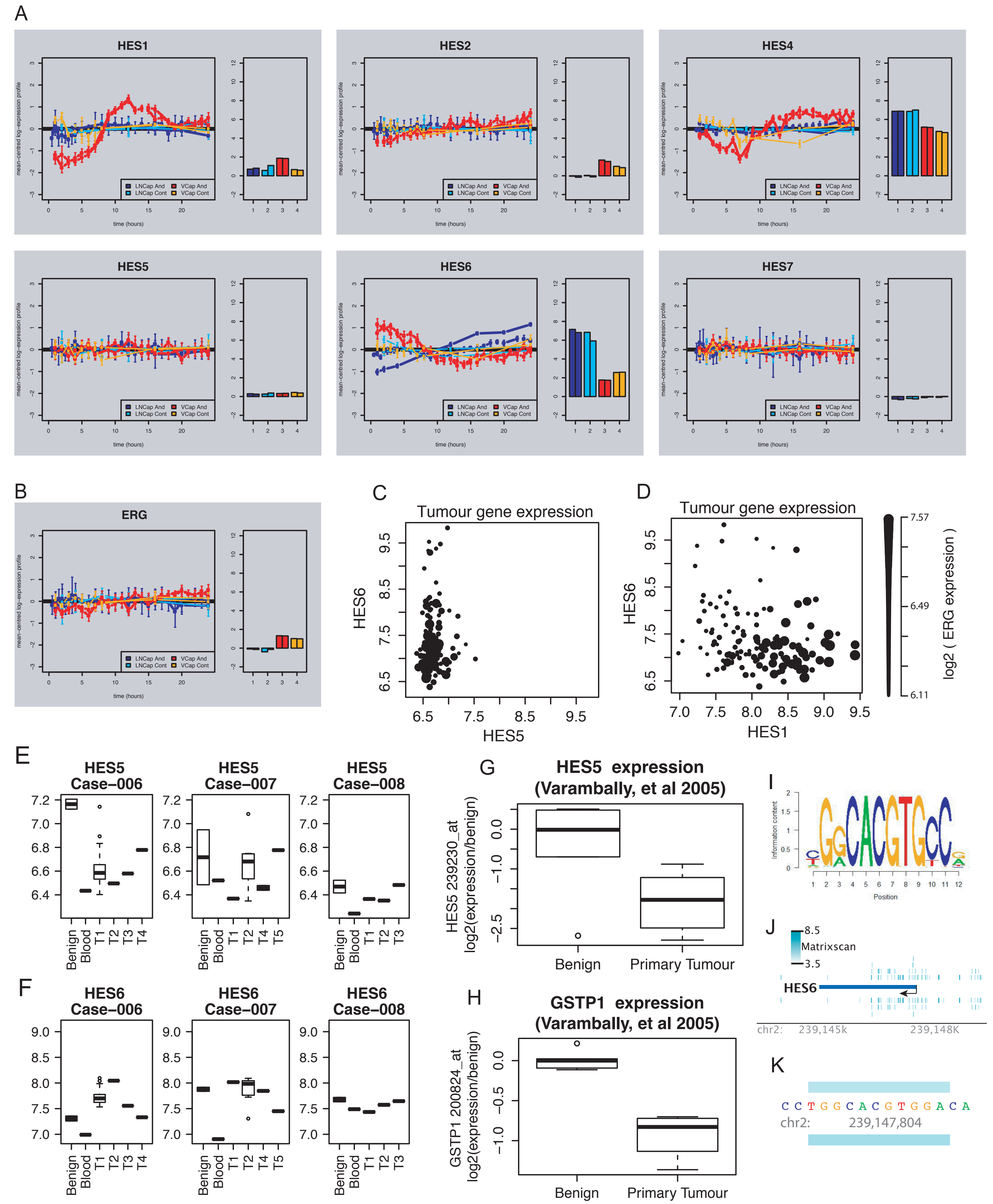

Supplementary Figure 5 Expression profiles of the HES gene family in response to androgen stimulation and in human tissue. (A-B) Time-course and bar plots of androgen stimulation gene expression time-series in LNCaP (ERG-VE) and VCaP (ERG+VE) prostate cancer cells, for (A) the HES gene family and (B) ERG. (C-D) Scatter plots of gene expression in human prostate tumours showing the relationship between (C) HES5 and HES6, (D) HES1 and HES6, points scaled by ERG expression. (E-F) Boxplot showing the expression of (E) HES5 and (F) HES6 in prostate tumour cores and adjacent benign tissue from the multifocal prostate cancer cohort (Supplementary Figure 1a). (G-H) Boxplots showing the expression of (G) HES5 and (H) GSTP1 in a separate cohort of benign and tumour prostate tissue (GSE3325). (I) HES5 consensus motif (SELEX derived), (J) RSAT matrixscan search using the HES5 motif at HES6 locus and (K) example f a HES5 binding sequence at the HES6 locus (visualised using BioSAVE).
