# Supplementary material for: HES5 silencing is an early and recurrent change in prostate tumourigenesis
Source: Endocr Relat Cancer. 2015 Jan 5;22(2):131–44. doi: 10.1530/ERC-14-0454 (PMC4335379; doi:10.1530/ERC-14-0454)
Supplement: Supplementary Figure [file supp_ERC-14-0454_Supplementary_figure_6.pdf]

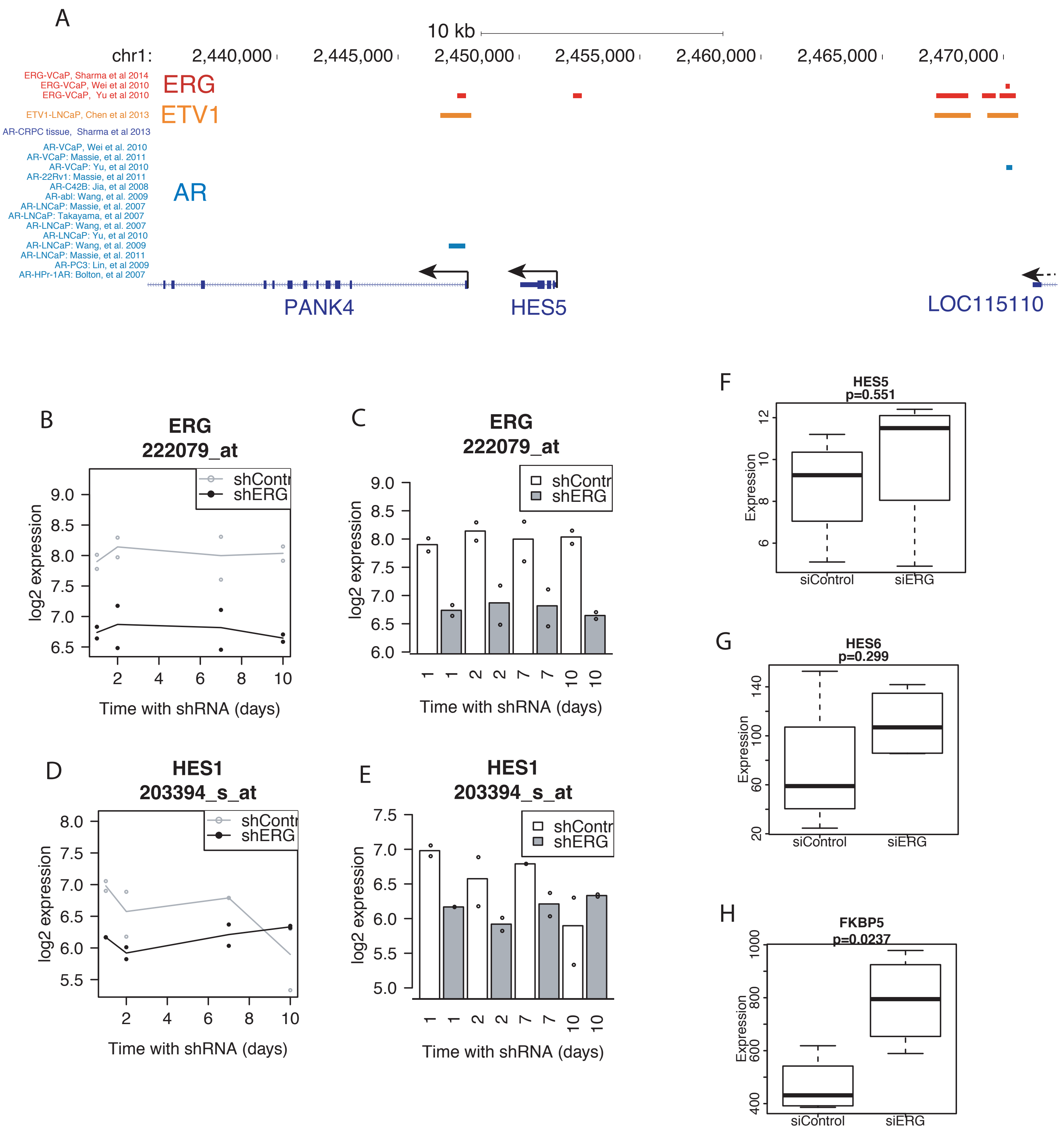

Supplementary Figure 6 Genomic binding profiles for ERG, ETV1 and the AR in prostate cells and pair-wise analysis of gene expression for key nodes in our predicted transcriptional network. (A) The HES5 gene locus, showing evidence of ERG and ETV1 binding ~20kb upstream of the HES5 gene and no convincing evidence of AR binding at this locus. A scale bar is shown at the top together with chromosomal locations and the HES5 gene location and orientations are indicated at the bottom. Where available multiple data sets are included for each factor, with the labelling scheme 'factor-sample, study' (i.e. 'AR-VCaP, Wei et al 2010' represent the binding profile of the AR in VCaP cells from the study of Wei, et al. 2010). (B-E) Transcript profiling from a doxycycline inducible ERG shRNA timecourse (with 24h, 48h, 7d and 10d timepoints;). Duplicate shRNA control and ERG knockdown are represented by grey and black points (respectively) for each timepoint. Data are represented as timecourse line-plots (B and D) and barplots (C and E) to show the profile with time and the pairwise comparisons. (F-H) Boxplots showing the expression of (F) HES5, (G) HES6 and (H) the classical AR target gene FKBP5 in control or ERG knock-down VCaP cells (GSE60771). Significance testing was performed using t-tests, p-values annotated on each plot.
