# Supplementary material for: HES5 silencing is an early and recurrent change in prostate tumourigenesis
Source: Endocr Relat Cancer. 2015 Jan 5;22(2):131–44. doi: 10.1530/ERC-14-0454 (PMC4335379; doi:10.1530/ERC-14-0454)
Supplement: Supplementary Figure [file supp_ERC-14-0454_Supplementary_figure_7.pdf]

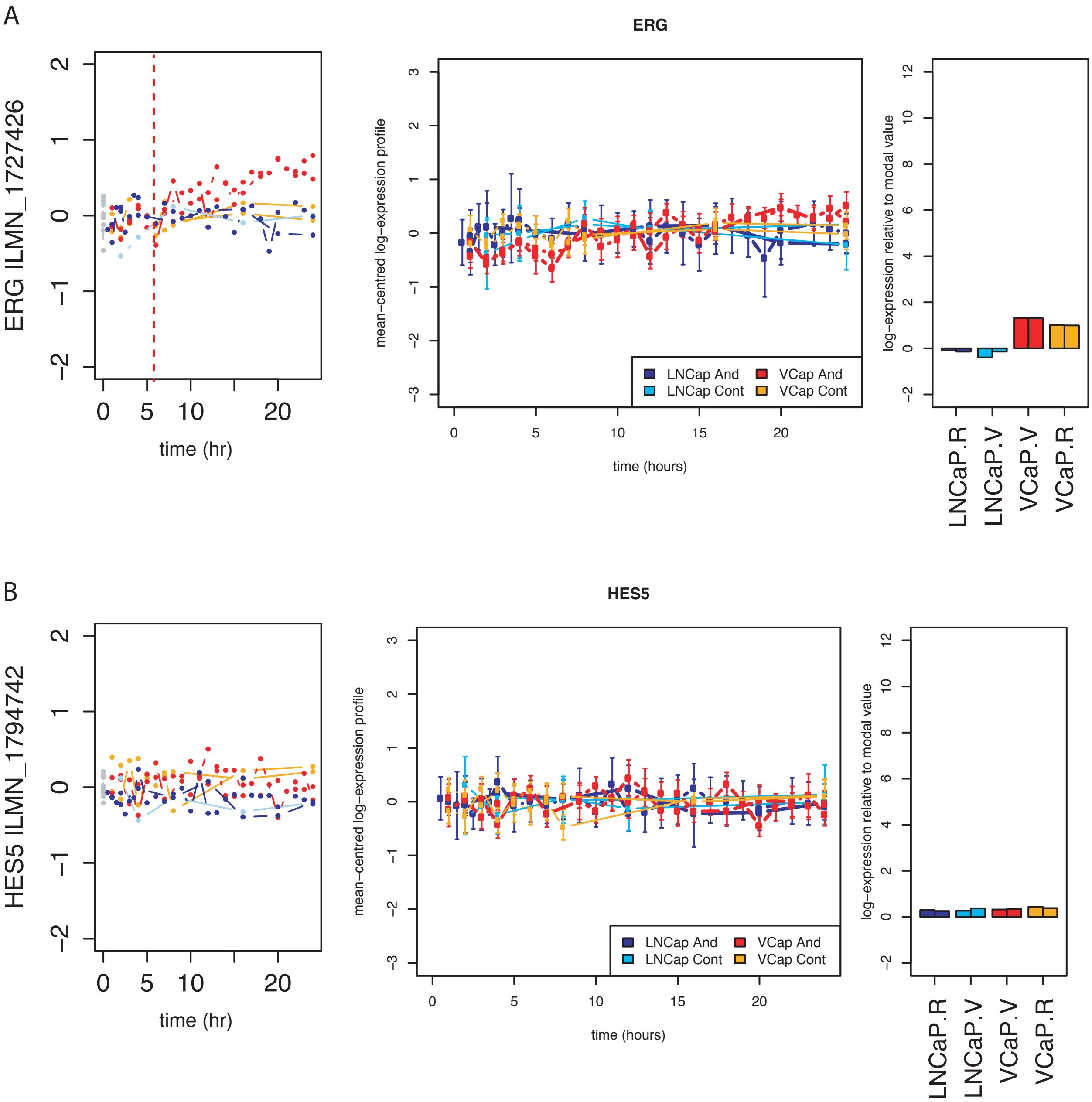

Supplementary Figure 7 Detailed gene expression timecourse analysis supports an AR-ERG-HES1-HES6 transcriptional cascade. (A-B) Androgen stimulation time-course gene expression profiles from VCaP (ERG-positve) and LNCaP (ERG-negative) prostate cancer cells. Panels on the left show the time-zero cenetred transcriptional profiles (log2 ratios), panels in the center show mean-centered transcript profiles (with s.e.m.) and panels on the right show bar plots of the mean expression levels (log2 intensity) for (A) ERG and (B) HES5. Data points are colour coded by condition and colour codes are consistent across all plots.
