# Supplementary material for: HES5 silencing is an early and recurrent change in prostate tumourigenesis
Source: Endocr Relat Cancer. 2015 Jan 5;22(2):131–44. doi: 10.1530/ERC-14-0454 (PMC4335379; doi:10.1530/ERC-14-0454)
Supplement: Supplementary Data [file supp_ERC-14-0454_Supplementary_information_1.pdf]

Supplementary Note

The UK Prostate ICGC Group consists of:

Colin Cooper[1,2,25], Rosalind Eeles[1,3,25], David C Wedge[4], Peter Van Loo[4,5], Gunes Gundem[4], Adam P Butler[4], Jilur Ghori[4], Jonathan D Kay[7], Hayley J Luxton[7], Steve Hawkins[7], Andy G Lynch[6], Sandra Edwards[1], Charlie E Massie[7], Daniel Leongamornlert[1], ZSofia Kote-Jarai[1], Nening Dennis[3], Sue Merson[1], Niedzica Camacho[1], Cathy Corbishley[8], Sarah Thomas[3], Sarah O'Meara[4], Lucy Matthews[1], Jeremy Clark[2], Christopher Greenman[15], Dan Berney[16], Steven Hazell[3], Naomi Livni[3], The ICGC Prostate Group, Cyril Fisher[3], Christopher Ogden[3], Pardeep Kumar[3], Alan Thompson[3], Christopher Woodhouse[3], David Nicol[3], Erik Mayer[3], Tim Dudderidge[3], Katalin Karaszi, [22] Adam Lambert[22], Clare Verrill[24], Luke Marsden[22], Yong Jie Lu[21], Freddie Hamdy[22], Steve Bova,[23], Andrew Futreal[4,25], Douglas Easton[17,25], Anne Y Warren[18], Christopher S Foster[19,25], Michael R Stratton[4,25], Hayley C Whitaker[7], Ultan McDermott[4,25], Daniel Brewer[1,2], David E Neal[7,20,25].

Affiliations

[1]Division of Genetics and Epidemiology, The Institute Of Cancer Research, Sutton, UK
[2]Also at Cancer Genetics, School of Biological Sciences and Norwich Medical School, University of East Anglia, Norwich, UK
[3]Royal Marsden NHS Foundation Trust, London and Sutton, UK
[4]Cancer Genome Project, Wellcome Trust Sanger Institute, Hinxton, UK
[5]Human Genome Laboratory, Department of Human Genetics, VIB and KU Leuven, Leuven, Belgium
[6]Statistics and Computational Biology Laboratory, Cancer Research UK Cambridge Research Institute, Cambridge, UK
[7] Uro-Oncology Research Group, Cancer Research UK Cambridge Institute, Cambridge, UK
[8]Department of Histopathology, St Georges Hospital, London, UK
[9]Institute of Food Research, Norwich Research Park, Norwich, UK
[10]Department of Medical Biophysics, University of Toronto, Toronto, Canada
[11]Department of Radiation Oncology, University of Toronto, Toronto, Canada
[12]Princess Margaret Cancer Centre-University Health Network, Toronto, Canada
[13]Informatics and Bio-Computing, Ontario Institute for Cancer Research, Toronto, Canada
[14]Department Pharmacology & Toxicology, University of Toronto, Toronto, Canada
[15]School of Computing Sciences, University of East Anglia, Norwich, UK
[16]Department of Molecular Oncology, Barts Cancer Centre, Barts and the London School of Medicine and Dentistry, London, UK
[17]Centre for Cancer Genetic Epidemiology, Department of Oncology, University of Cambridge, Cambridge, UK
[18]Department of Histopathology, Cambridge University Hospitals NHS Foundation Trust, Cambridge, UK
[19]University of Liverpool and HCA Pathology Laboratories, London, UK
[20]Department of Surgical Oncology, University of Cambridge, Addenbrooke's Hospital, Cambridge, UK
[21]Centre for Molecular Oncology, Barts Cancer Institute, Queen Mary University of London, London, UK.
[22]Nuffield Department of Surgical Sciences, University of Oxford, Old Road Campus Research Building, Oxford OX3 7DQ, UK
[23]Institute of Biosciences and Medical Technology-BioMediTech, University of Tampere and Tampere University Hospital, Tampere, Finland
[24]Department of Cellular Pathology, John Radcliffe Hospital, Oxford, UK
[25]Senior Principal Investigators of the Cancer Research UK funded ICGC Prostate Cancer Project
